# Distinct Molecular Responses to Ketamine and Imipramine in Cortical and Striatal Regions Following Acute Swim Stress

**DOI:** 10.3390/biom16040484

**Published:** 2026-03-24

**Authors:** Veronica Begni, Floriana De Cillis, Natascha Pfeiffer, Steven Roger Talbot, Peter Gass, Annamaria Cattaneo, Marco Andrea Riva, Anne Stephanie Mallien

**Affiliations:** 1 Biological Psychiatry Unit, IRCCS Istituto Centro San Giovanni di Dio Fatebenefratelli, 25125 Brescia, Italy; vbegni@fatebenefratelli.eu (V.B.); annamaria.cattaneo@unimi.it (A.C.); m.riva@unimi.it (M.A.R.); 2 Department of Pharmacological and Biomolecular Sciences, University of Milan, 20122 Milan, Italy; floriana.decillis@unimi.it; 3 RG Animal Models in Psychiatry, Department of Psychiatry and Psychotherapy, Central Institute of Mental Health, Medical Faculty Mannheim, Heidelberg University, 68159 Mannheim, Germany; natascha.pfeiffer@zi-mannheim.de (N.P.); peter.gass@zi-mannheim.de (P.G.); 4 Institute for Laboratory Animal Science, Hannover Medical School, 30625 Hannover, Germany; talbot.steven@mh-hannover.de; 5 German Center for Mental Health (DZPG), Partner Site Mannheim, Heidelberg, Ulm, Germany

**Keywords:** ketamine, imipramine, immediate early genes, swim stress, coping

## Abstract

Pharmacological antidepressant treatments alter the molecular and functional reactivity of stress-sensitive neural networks. However, how classical versus rapid-acting antidepressants differentially modulate acute stress-induced transcriptional responses across brain regions remains unclear. Here, we compared imipramine and ketamine in mice exposed to acute swim stress, assessing transcriptional adaptations across the frontal cortex, hippocampus, and striatum. Swim stress induced significant widespread activation of *cFOS*, which led to drug-specific modulations: imipramine primarily significantly dampened cortical and striatal *cFOS* expression, whereas ketamine preserved stress-evoked neuronal activation. In contrast, hippocampal activation was significantly robust but largely unaffected, indicating that acute antidepressant drug effects during stress coping preferentially target cortical and striatal plasticity mechanisms. In contrast, *BDNF* expression was altered only within the striatal region, where imipramine attenuated the stress-related increase in *BDNF* expression. Statistical analysis of behavioral outcomes during the swim stress confirmed a shared facilitation of active coping, yet these similar outcomes emerged from distinct molecular programs. Together, the data demonstrate that the treatment effects of the two substances diverge mechanistically, revealing cortical and striatal transcriptional signatures of classical versus rapid-acting antidepressant action. While these findings suggest potential translational relevance for understanding distinct mechanisms, further studies in humans are required to validate these signatures and their clinical implications.

## 1. Introduction

Major depressive disorder (MDD) is a leading cause of disability worldwide and remains a major public health burden despite the availability of several classes of antidepressant drugs [1]. Classical antidepressants are effective in a subset of patients but typically require weeks to months of continuous administration before clinical improvement emerges [1]. This therapeutic delay, together with a high proportion of treatment-resistant cases, underscores the urgent need for novel strategies targeting alternative mechanisms of action [1].

Ketamine, a non-competitive N-methyl-D-aspartate (NMDA) receptor antagonist, has received considerable attention in recent years due to its ability to elicit rapid antidepressant effects [1]. Preclinical studies in rodents have shown that ketamine exerts rapid and sustained antidepressant-like effects across several stress-based paradigms, including chronic unpredictable stress, chronic social defeat stress, and corticosterone-induced models, where it reverses behavioral deficits [2]. Moreover, such studies have demonstrated that ketamine promotes synaptogenesis and enhances synaptic plasticity in the prefrontal cortex and hippocampus, effects that are thought to be mediated through a surge in glutamate release and activation of AMPA receptors, ultimately leading to increased Brain-Derived Neurotrophic Factor (BDNF) signaling and mTOR pathway activation [3]. Recent clinical studies confirmed ketamine’s efficacy in the treatment of MDD [4,5,6].

Investigating how antidepressants differentially regulate these transcriptional programs across stress-responsive brain regions may provide novel insight into their mechanisms of action. Therefore, in addition to the pharmacological treatment, we exposed the mice to swim stress, as a robust molecular trigger that shapes the transcriptional landscape [7]. We analyzed the stress coping behavior during the swim stress to confirm that the two drug treatments elicited comparable positive coping responses. This response provides an opportunity to investigate molecular signatures and their modulation by pharmacological treatments. For instance, immediate early genes (IEGs) such as *Arc*, *Npas4*, *Zif268*, and *c-Fos* represent sensitive markers of neuronal activation and synaptic plasticity, whereas BDNF is a key mediator of longer-term neuroplastic adaptations [8,9,10].

Here, we performed a comparative analysis of ketamine and imipramine in male C57BL/6N mice subjected to swim stress [11]. By integrating behavioral measures with region-specific expression of IEGs and BDNF in the frontal cortex, hippocampus, and striatum, our goal was to capture both the phenotypic and molecular signatures associated with drug-modulated stress coping responses. This approach allowed us to identify convergent and divergent patterns of action between ketamine and imipramine, highlighting potential neural substrates underlying their distinct clinical profiles.

## 2. Materials and Methods

### 2.1. Animals and Experimental Design

Forty-two male C57BL/6N mice (Charles River Laboratories, Sulzfeld, Germany) were used. At the start of the study, animals were 8 weeks old, weighed 18–25 g, and were housed individually in type II cages containing nesting tissue and wooden aspen chips as bedding, with food and water provided ad libitum. Environmental conditions were kept constant (12 h light/dark cycle, lights off at 07:00 a.m.; temperature 22.1 ± 1 °C; humidity 45 ± 5%). All procedures were approved by the Regierungspräsidium Karlsruhe (35-9185-81-G-102-18) and conducted in compliance with Directive 2010/63/EU of the European Parliament. The sample size was determined a priori using a biometric power analysis performed with G*Power (version 3.1.9.2). Unfortunately, one mouse was excluded from the study due to health issues unrelated to the experimental procedures before the experimental onset.

Animals were randomly assigned to three treatment groups: vehicle (saline, *n* = 13), ketamine (10 mg/kg, *n* = 14), or imipramine (20 mg/kg, *n* = 14). Ketamine ((±)-ketamine hydrochloride, catalog number: K2753, Sigma-Aldrich, St. Louis, MO, USA) was dissolved in saline at 2 mg/mL, and imipramine (imipramine hydrochloride, catalog number: I0899, Sigma-Aldrich) in saline at 4 mg/mL. Animals were randomized in two steps. First, upon arrival, animals were randomly assigned to positions within the cage racks to minimize environmental effects. Second, animals were stratified by body weight and then randomly allocated to experimental groups to ensure comparable average weights across groups. To ensure precise timing, a maximum of three animals were handled concurrently. Each animal received an intraperitoneal injection of either vehicle, ketamine or imipramine, and the 30 min post-injection interval was individually tracked for each animal. After this interval, animals were either subjected to 6 min swim stress (saline, *n* = 6; ketamine, *n* = 7; imipramine, *n* = 7) or left undisturbed in their home cages (saline, *n* = 7; ketamine, *n* = 7; imipramine, *n* = 7). Swim stress was performed in individual glass beakers (1 L water, 21 °C) positioned in a stand that prevented visual contact between mice and allowed video recording of behavior. Up to three animals were swam in parallel, with the order of injection and swim stress randomized across treatment groups. Following swim stress, animals were dried under a red light for 10 min to allow recovery without additional handling stress, then killed 20 min later. Animals that did not swim were killed at the same post-injection interval as their swim-stressed counterparts, ensuring time-matching for subsequent IEG/BDNF analyses. The total experimental period ran for 5 h, with all interventions matched individually, ensuring stress exposure and sampling were precisely controlled across treatment and control groups. All mice were killed by cervical dislocation (Supplementary Figure S1). Brains were rapidly extracted, dissected on ice and frozen on dry ice. Specifically, the frontal cortex, hippocampus, and striatum were collected and stored at –80 °C for subsequent gene expression analyses.

### 2.2. Swim Stress and Coping Analysis

The swim stress was conducted in a 2 L glass beaker (24 cm height, 12 cm outer diameter, 10 cm inner diameter) filled with 1 L of water maintained at 21 °C. The session was 6 min for each mouse. Active and passive coping responses to the swim stress were assessed as immobility time and latency to immobility. Following swim stress, animals were dried under a red light for 10 min to allow recovery without additional handling stress.

### 2.3. Quantitative Real-Time PCR (qRT-PCR)

Total RNA was isolated from the frontal cortex, hippocampus, and striatum using PureZol RNA isolation reagent (Bio-Rad Laboratories, Segrate, Italy) according to the manufacturer’s protocol. Samples were treated with DNase to remove residual genomic DNA. RNA concentration and purity were determined using a NanoDrop spectrophotometer (Thermo Fisher, Wilmington, DE, USA), and samples were diluted to 10 ng/μL for qRT-PCR.

qRT-PCR was performed on a CFX384 real-time system (Bio-Rad Laboratories, Segrate, Italy) using the iScript™ One-Step RT-PCR Kit for Probes (Bio-Rad Laboratories, Segrate, Italy). Reactions were run in triplicate in 384-well plates. Probes and primers for *Arc* (Mm00479619_g1) and *Gapdh* (Mm99999915_g1) were obtained from Thermo Fisher Scientific(Waltham, MA, USA), while additional TaqMan assays were purchased from Eurofins Genomics (Vimodrone, Italy) (see Supplementary Table S1). *Gapdh* served as the endogenous control. Data was analyzed using an efficiency-corrected model accounting for amplification efficiencies of target and reference genes [12]. Results were expressed as fold change relative to the control group (set to 100%).

To obtain a composite measure of immediate early gene (IEG) activation, individual z-scores were computed for each of four IEGs (*Arc, Zif268, Npas4, and c-Fos*) per animal, brain region, and experimental condition. For each gene within each brain region, expression values were standardized to the vehicle/no-stress control group (z = [x − $M_{v\mathrm{EH}}/{\mathrm{SD}}_{v\mathrm{EH}}$). The IEG z-score for each animal was then calculated as the arithmetic mean of the four gene-level z-scores, yielding a single composite index of IEG induction per region and condition. BDNF was not included in this composite because it is a neurotrophin rather than an activity-dependent IEG.

### 2.4. Statistical Analysis

The experimental unit was the individual animal. All animals were included in the study except for one mouse, which was excluded due to health issues unrelated to the experimental procedures. No other animals met any exclusion criteria. For molecular analyses, not all samples could be included due to technical issues during RNA extraction. Additionally, outliers in the qPCR data were identified and excluded using SPSS (version 30). Therefore, the number of biological replicates differs between behavioral and gene expression datasets. The exact *n* for each experimental condition is reported at the base of the bars in every figure.

Data are presented as mean ± SEM. Statistical analyses were performed using GraphPad Prism 8 (GraphPad Software, San Diego, CA, USA) and SPSS Statistics 29.0.0 (IBM, Armonk, NY, USA). Data were analyzed using R 4.5.1 with the rstatix and effectsize packages, as well as GraphPad Prism 8 for additional visualization.

Behavioral data were analyzed using one-way ANOVA (Type II) with drug as a between-subject factor and latency to immobility or total immobility time as dependent variables. Significant main effects were further explored with Tukey’s HSD post hoc tests to assess pairwise differences between groups.

Effect sizes for all pairwise comparisons were calculated using Cohen’s d along with 95% confidence intervals to quantify the magnitude and uncertainty of the effects. Statistical significance for pairwise comparisons was determined using Tukey-adjusted *p*-values (α = 0.05). Cohen’s d and its 95% confidence intervals are reported as descriptive measures of effect size.

Gene expression data for five neuroplasticity-related markers (*BDNF*, *Arc*, *Zif268*, *Npas4*, and *c-Fos*) were analyzed across three brain regions (frontal cortex [FC], hippocampus [HIPP], and striatum [STR]) in a 3 × 2 between-subjects factorial design, with drug treatment (vehicle [VEH], ketamine [KET], imipramine [IMI]) and stress condition (no stress vs. stress) as fixed factors. Because the same animals contributed tissue from all three brain regions and all five genes were measured within each region, gene and region were treated as within-subject factors.

Data were analyzed using a linear mixed-effects model (LMM) fitted with restricted maximum likelihood (REML) estimation using the lme4 package (v1.1-35) [13] in R (v4.4.1) [14]. The fixed-effects structure included the full factorial interaction of treatment, stress, gene, and region (Treatment × Stress × Gene × Region). A random intercept for subject was included to account for the repeated-measures dependency across genes and brain regions within each animal. The model was specified as: Value ~ Treatment × Stress × Gene × Region + (1|Subject). Type III F-tests with Satterthwaite’s approximation for denominator degrees of freedom were obtained via the lmerTest package [15]. Partial eta-squared () was computed for each fixed effect as SSeffect/(SSeffect + SSresidual).

Post hoc pairwise contrasts were derived from estimated marginal means (EMMs) using the emmeans package Version 2.0.2 [16]. Two families of contrasts were computed: (a) stress contrasts (no stress vs. swim stress) within each treatment level, separately for each gene × region combination (3 treatments × 5 genes × 3 regions = 45 contrasts), and (b) treatment contrasts (all pairwise comparisons among VEH, KET, and IMI) within each stress condition, separately for each gene × region combination (3 pairs × 2 stress levels × 5 genes × 3 regions = 90 contrasts). All 135 contrasts were corrected for multiple comparisons using the Benjamini–Hochberg (BH) procedure [17], which controls the false discovery rate (FDR) across the full set of tests. Cohen’s d was computed for each pairwise comparison using pooled standard deviations from the raw data. Ninety-five percent confidence intervals for Cohen’s d were computed using the noncentral t-distribution method [18], as implemented in the effectsize package for R [19]. Statistical significance was set at α = 0.05 (two-tailed) for all tests.

The composite IEG z-scores were analyzed separately for each brain region (frontal cortex [FC], hippocampus [HIPP], and striatum [STR]). Within each region, the a priori contrast of interest was the effect of swim stress (no stress vs. forced swim test [FST]) within each treatment group (VEH, KET, IMI), yielding three comparisons per region and nine comparisons in total. Welch’s *t*-tests were used to accommodate unequal group sizes and potential variance heterogeneity. All nine *p*-values were corrected for multiple comparisons using the Benjamini–Hochberg (BH) procedure. Cohen’s d with pooled standard deviations was computed for each contrast, and 95% confidence intervals were obtained via the noncentral t-distribution. Statistical significance was set at α = 0.05 (two-tailed).

Model assumptions were evaluated by inspecting residual diagnostics. Normality of residuals was assessed using Q–Q plots and Shapiro–Wilk tests, and homoscedasticity was examined using residuals versus fitted values plots. No substantial deviations from these assumptions were detected. Data transformations were considered but were not required.

## 3. Results

### 3.1. Ketamine and Imipramine Induce Active Coping During Swim Stress

**Figure 1:**
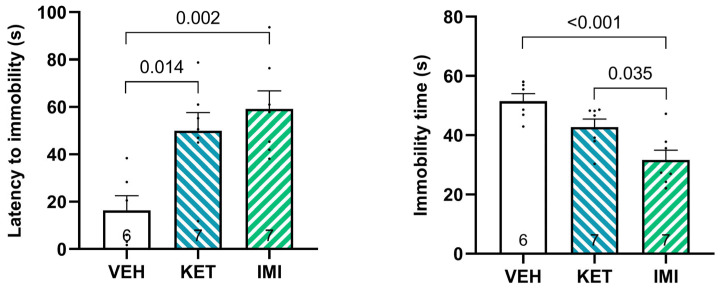
Analysis of the latency time to first immobility and the total immobility time of mice who were pre-treated with vehicle, ketamine, or imipramine and exposed to swim stress. Data are expressed as the mean ± SEM. Number of samples is indicated at the base of the bars. Significant results from comparisons are indicated by displaying the *p*-values.

To assess the behavioral effects of ketamine and imipramine, we measured two complementary parameters during swim stress: latency to immobility and total immobility time (Figure 1).

A significant main effect of pharmacological treatment was detected for latency to immobility (F(2, 17) = 9.04, *p* = 0.002, η^2^ = 0.515), with post hoc comparisons revealing that both ketamine (*p* = 0.014) and imipramine (*p* = 0.002) significantly increased latency compared to vehicle-treated mice. The magnitude of these effects was large (ketamine: Cohen’s d = 1.85, 95% CI [0.49–3.15]; imipramine: Cohen’s d = 2.38, 95% CI [0.88–3.82]). The comparison between ketamine and imipramine was not significant (*p* = 0.637, d = 0.46, 95% CI [−0.62–1.51]).

Total immobility time was also significantly affected by treatment (F(2, 17) = 11.32, *p* = 0.000749, η^2^ = 0.571), and post hoc analysis showed that imipramine markedly reduced immobility compared to both vehicle (*p* = 0.000543) and ketamine (*p* = 0.0345). The magnitude of these effects was large (vs. vehicle: Cohen’s d = −2.55, 95% CI [−4.03–−1.00]; vs. ketamine: Cohen’s d = −1.38, 95% CI [−2.54–−0.17]). In contrast, ketamine did not significantly alter immobility relative to vehicle.

### 3.2. Effects of Ketamine and Imipramine on Gene Expression

**Figure 2:**
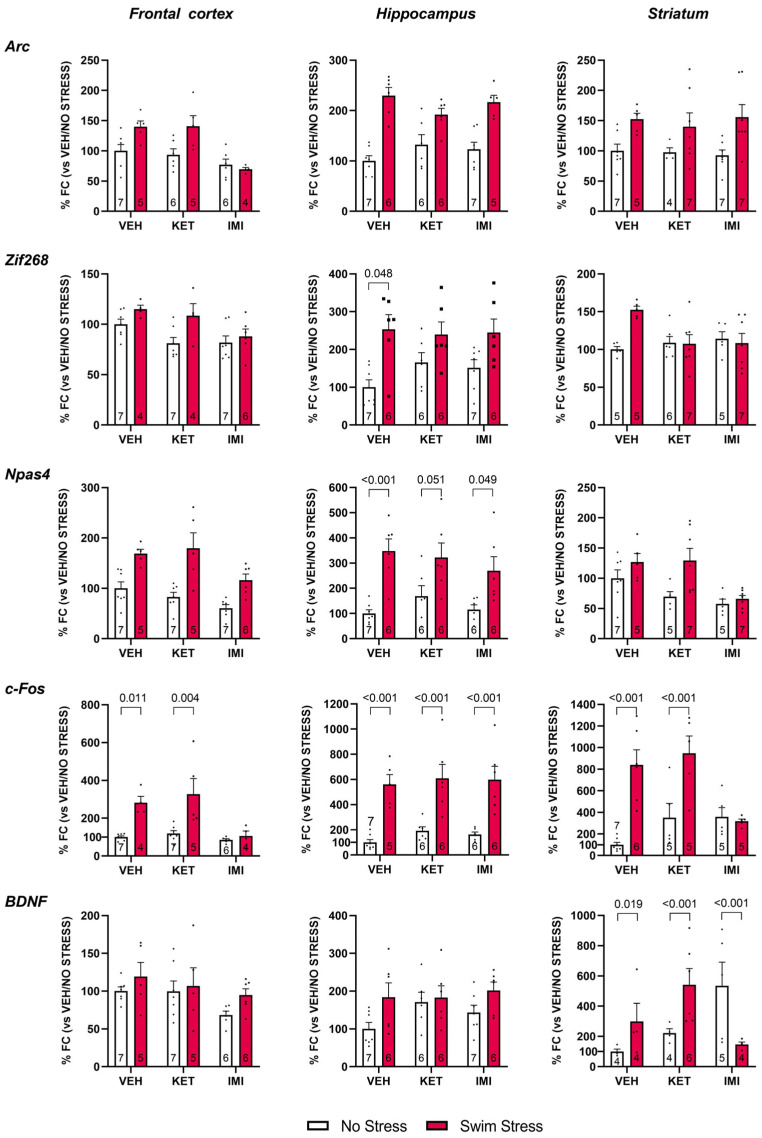
Analysis of *Arc*, *Zif268*, *Npas4, c-Fos,* and *BDNF* mRNA levels in the brain of mice who were pre-treated with vehicle, ketamine, or imipramine and exposed or not to swim stress. Data are expressed as the mean ± S.E.M. of vehicle-treated mice not exposed to the stress, set as 100%. Number of samples is indicated at the base of the bars. Significant results from comparisons are indicated by displaying the *p*-values.

Next, we aimed to determine how pretreatment with imipramine or ketamine modulates the transcriptional responses elicited by stress exposure. To this end, we analyzed the modulation of activity-dependent genes in the frontal cortex, hippocampus, and striatum, regions critically involved in stress processing and antidepressant action, by comparing animals pretreated with vehicle, imipramine, or ketamine. Given the rapid and transient nature of immediate early gene (IEG) expression, we focused on *Arc*, *Npas4*, *Zif268*, and *c-Fos*, which are known to be induced within 30–60 min after neuronal activation [20,21,22,23,24,25], a time window that aligns with our experimental design. This approach allowed us to capture the region- and gene-specific molecular signatures associated with swim stress and their modulation by pharmacological treatments (Figure 2).

The analysis included 528 observations from 41 subjects. Degrees of freedom were estimated using the Satterthwaite approximation. Effect sizes are reported as partial eta squared (η^2^p).

A total of 528 observations from 41 subjects were included in the analysis (see Table 1). The omnibus LMM revealed significant main effects of FST (F(1, 31.4) = 73.15, *p* < 0.001, η^2^p = 0.149), gene (F(4, 403.6) = 85.39, *p* < 0.001, η^2^p = 0.450), region (F(2, 412.5) = 64.95, *p* < 0.001, η^2^p = 0.237), and treatment (F(2, 31.4) = 4.40, *p* = 0.021, η^2^p = 0.021). Importantly, several interactions were significant. The Treatment × FST interaction was significant (F(2, 31.4) = 8.54, *p* = 0.001, η^2^p = 0.039), indicating that treatment effects differed across stress conditions. The FST × Gene interaction (F(4, 403.6) = 42.05, *p* < 0.001, η^2^p = 0.287) and the Gene × Region interaction (F(8, 404.4) = 22.75, *p* < 0.001, η^2^p = 0.304) were also highly significant, reflecting that stress effects and regional expression patterns varied across genes. Higher-order interactions, including the four-way Treatment × FST × Gene × Region interaction (F(16, 404.4) = 4.51, *p* < 0.001, η^2^p = 0.147), were also significant, indicating that the interplay between drug treatment and stress in gene expression was both gene- and region-specific.

BH-corrected post hoc contrasts identified 23 significant pairwise comparisons out of 135 tested. The most robust FST effects were observed in the hippocampus and striatum. In the hippocampus, swim stress markedly increased *c-Fos* expression in all three treatment groups (VEH: d = −3.89, 95% CI [−5.94, −1.83], *p* < 0.001; KET: d = −2.09, 95% CI [−3.54, −0.63], *p* < 0.001; IMI: d = −2.33, 95% CI [−3.85, −0.80], *p* < 0.001) and *Npas4* expression in the VEH (d = −2.93, 95% CI [−4.57, −1.29], *p* < 0.001) and IMI (d = −1.52, 95% CI [−2.83, −0.21], *p* = 0.049) groups. Hippocampal *Zif268* was also significantly elevated by swim stress in VEH-treated animals (d = −2.05, 95% CI [−3.43, −0.66], *p* = 0.048). In the striatum, swim stress strongly increased *c-Fos* in both VEH (d = −3.13, 95% CI [−4.83, −1.43], *p* < 0.001) and KET groups (d = −1.82, 95% CI [−3.34, −0.29], *p* < 0.001). Striatal *BDNF* showed divergent stress effects across treatments: swim stress increased BDNF in VEH (d = −1.17, 95% CI [−2.71, 0.36], *p* = 0.019) and KET (d = −1.53, 95% CI [−3.00, −0.06], *p* < 0.001) groups but decreased it in the IMI group (d = 1.47, 95% CI [−0.05, 3.00], *p* < 0.001). In the frontal cortex, swim stress significantly increased *c-Fos* expression in VEH (d = −4.40, 95% CI [−6.78, −2.03], *p* = 0.011) and KET (d = −1.74, 95% CI [−3.12, −0.37], *p* = 0.004) groups.

Significant between-treatment contrasts were concentrated in the striatum and frontal cortex. In the striatum under no-stress conditions, both KET and IMI groups showed higher *BDNF* than VEH (VEH vs. KET: d = −0.96, 95% CI [−2.31, 0.39], *p* = 0.006; VEH vs. IMI: d = −1.65, 95% CI [−3.22, −0.08], *p* < 0.001; KET vs. IMI: d = −1.18, 95% CI [−2.63, 0.27], *p* < 0.001). Under swim stress, striatal *BDNF* was significantly higher in KET than IMI (d = 1.91, 95% CI [0.33, 3.48], *p* < 0.001). For *c-Fos* in the striatum, VEH-treated animals showed lower expression than KET (d = −1.30, 95% CI [−2.58, −0.02], *p* < 0.001) and IMI (d = −1.99, 95% CI [−3.43, −0.55], *p* < 0.001) under no-stress conditions, whereas under swim stress, IMI showed markedly lower *c-Fos* than VEH (d = 2.02, 95% CI [0.51, 3.53], *p* < 0.001) and KET (d = 2.46, 95% CI [0.73, 4.19], *p* < 0.001). In the frontal cortex under swim stress, IMI-treated animals had lower *c-Fos* expression than both VEH (d = 2.91, 95% CI [0.76, 5.06], *p* = 0.048) and KET (d = 1.56, 95% CI [0.01, 3.10], *p* = 0.011).

**Figure 3:**
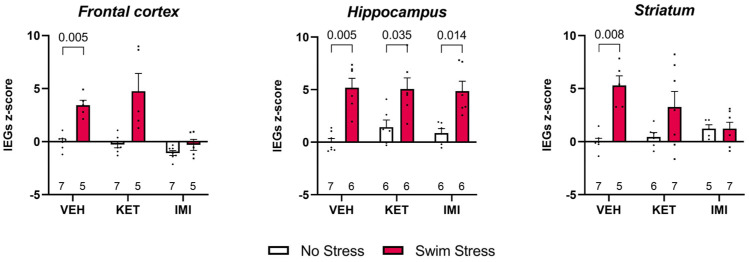
Analysis of IEG z-scores in the frontal cortex, hippocampus, and striatum of mice who were pre-treated with vehicle, ketamine, or imipramine and exposed or not to swim stress. Data are expressed as the mean ± S.E.M. of vehicle-treated mice not exposed to the swim stress. The number of samples is indicated at the base of the bars. Significant results from comparisons are indicated by displaying the *p*-values.

Composite IEG z-scores were computed from 110 observations across three brain regions. Descriptive statistics and individual data points are presented in Figure 3.

In the hippocampus, swim stress significantly elevated the composite IEG response in all three treatment groups (VEH: d = −3.20, 95% CI [−4.89, −1.46], *p* = 0.006; KET: d = −1.66, 95% CI [−2.97, −0.29], *p* = 0.035; IMI: d = −2.23, 95% CI [−3.69, −0.71], *p* = 0.014). This indicates a robust, treatment-independent stress-induced IEG activation in HIPP.

In the frontal cortex, a significant stress effect was observed for VEH-treated animals (d = −3.95, 95% CI [−5.97, −1.86], *p* = 0.005). The KET group showed a numerically large effect that did not survive BH correction (d = −2.05, 95% CI [−3.46, −0.57], *p* = 0.059), likely due to high inter-individual variability in the swim-stress condition (SD = 3.76). No significant stress effect was found for IMI (d = −0.89, *p* = 0.251).

In the striatum, VEH-treated animals again showed a significant stress-induced increase in IEG composite scores (d = −3.68, 95% CI [−5.62, −1.69], *p* = 0.008). Neither KET (d = −0.96, *p* = 0.136) nor IMI (d = −0.01, *p* = 0.989) groups showed significant stress effects, suggesting that both drug treatments attenuated the stress-induced IEG response in this region.

Taken together, swim stress reliably induced composite IEG expression across all three brain regions in vehicle-treated animals. In the hippocampus, this effect persisted regardless of treatment, whereas in the frontal cortex and striatum, ketamine and imipramine appeared to attenuate the stress-induced IEG response.

## 4. Discussion

The present study used molecular analyses to investigate the acute effects of imipramine and ketamine in swim stress-exposed mice. We connected the observed coping strategies as behavioral readouts with the expression of IEGs (Arc, Npas4, Zif268 and c-Fos) and the neurotrophin BDNF across multiple brain regions. Our aim was to capture both the phenotypic and transcriptional signatures underlying coping responses and their modulation by pharmacological treatments.

Throughout this study, we discuss swim stress and coping rather than labeling behaviors as depressive-like. This is important to mention as the swim stress protocol follows a very common form of the forced swim test (FST). Our choice of this description reflects the growing recognition that the FST measures stress-coping strategies rather than mood per se [26,27]. In the past, many studies have misinterpreted the FST, equating immobility to despair. Throughout the literature, ‘despair’ is often used colloquially to describe prolonged immobility or passive coping in the FST. We emphasize that this term does not represent a discrete clinical symptom but rather a descriptive label for behaviors associated with stress-induced resignation or decreased active coping. Increasing evidence suggests that immobility does not index behavioral despair but may instead represent an adaptive, energy-conserving strategy [26,27]. By adjusting our reporting, we aim to clarify the behavioral meaning of the measures, without altering the established protocol itself. Water temperature is a critical factor influencing both physiological and behavioral responses during forced swimming [28]. Recent work emphasizes that the FST paradigm should be interpreted as a multifactorial stressor, combining elements of inescapability with physiological challenges such as immersion and heat loss [29].

Experimental studies show that swimming in relatively cold water induces pronounced reductions in core body temperature in rodents, confirming that thermoregulatory processes contribute to the overall stress response. Consistent with systematic welfare assessments, transient hypothermia represents the most consistent physiological effect of the test, while other long-lasting indicators of distress are limited [30].

Importantly, in our study all stressed animals were exposed to identical water temperature, test duration, and standardized recovery procedures, e.g., drying and warming. Therefore, although thermal effects contribute to the overall stress response, they cannot account for the observed between-group differences.

Here we found that both ketamine and imipramine significantly increased latency to immobility, indicating a shared ability to delay the transition from active to passive coping strategies under acute stress. While imipramine significantly reduced total immobility time, ketamine produced a smaller effect that did not reach statistical significance. These findings suggest only a partial overlap in the behavioral effects of the two treatments. The dissociation between the two behavioral measures is noteworthy: latency to immobility appears to reflect the threshold for disengaging from active coping, whereas total immobility indexes sustained passive coping capacity [26,27].

At the molecular level, we found that swim stress elicited a robust induction of IEGs across the frontal cortex, hippocampus, and striatum, reflecting a widespread engagement of multiple stress-sensitive brain regions. Furthermore, the observed IEG induction may be interpreted as a molecular signature of neuronal engagement as IEGs are established molecular correlates of neuronal activity and early synaptic plasticity [8]. Importantly, their dynamics of induction align with our experimental design, as these genes typically peak within 30–60 min after stimulation [20,21,22,23,24,25]. Notably, the time point we chose captures the immediate transcriptional response to stress as well as to drug exposure and therefore reflects early neural activation events rather than delayed antidepressant-like effects. This is particularly relevant for ketamine, whose rapid pharmacodynamic actions precede its longer-lasting antidepressant outcomes. Thus, our data primarily reflect the acute molecular impact of ketamine and imipramine in the context of stress, rather than adaptive processes. Accordingly, swim stress is likely the primary driver of IEG upregulation, while pharmacological treatments modulate the magnitude of this molecular response.

Specifically, drug treatments exerted region- and gene-specific modulatory effects. Imipramine attenuated the swim stress-induced increase in c-fos expression in the striatum and frontal cortex, but not in the hippocampus. In contrast, ketamine did not significantly affect c-fos stress-induced expression in any brain region. The only modulatory ketamine-related effect on IEGs was observed for Zif268 in the hippocampus, which showed a similar pattern following imipramine administration, in contrast to vehicle-treated animals. The effect of ketamine on Npas4 remains instead inconclusive, as the comparison between stress and no-stress conditions did not reach statistical significance (*p* = 0.051), although it may indicate a trend. Overall, ketamine reduced stress-induced Zif268 expression in the hippocampus and may have attenuated Npas4 responses in this region. By contrast, imipramine attenuated stress-induced c-fos expression in the frontal cortex and striatum and reduced Zif268 expression in the hippocampus.

Our results highlight how ketamine and imipramine, despite showing partially overlapping behavioral effects, suggest differential engagement of molecular pathways when administered acutely. Ketamine, through its well-characterized NMDA receptor antagonism, rapidly enhances glutamatergic transmission by disinhibiting pyramidal neurons. This excitatory surge promotes AMPA receptor activation and downstream signaling cascades such as BDNF–TrkB and mTORC1, which are central to synaptogenesis and plasticity [31]. In line with this, we observed that ketamine-treated animals preserved or even enhanced the induction of cFOS in response to stress, consistent with the possibility that ketamine interacts with the stress-induced wave of neuronal activation to facilitate processes related to synaptic plasticity. However, our data do not allow causal inference, and this interpretation remains speculative. This is coherent with the clinical profile of ketamine, where rapid antidepressant effects emerge within hours, likely due to its capacity to acutely unlock plasticity mechanisms [31]. Imipramine, in contrast, is a tricyclic antidepressant and requires chronic administration for clinical efficacy [1]. Its acute behavioral effect in our study potentially arises from a rapid increase in monoamines, which can transiently alter arousal and coping responses to stress. The suppression of cFOS induction observed in the cortex and striatum of imipramine-treated animals supports this interpretation. Rather than promoting cFOS expression in the acute window, imipramine appears to dampen stress-induced neuronal activation, potentially through the activation of inhibitory receptors, such as 5-HT1A autoreceptors in the raphe nuclei or postsynaptic 5-HT1A and α2-adrenergic receptors in target regions, resulting in reduced neuronal firing [32]. Such acute inhibition may serve to limit excitotoxicity or maladaptive neuronal dysregulation, preceding the adaptive changes induced by chronic treatment, including desensitization of inhibitory receptors and increased neuronal activity associated with therapeutic efficacy. However, given the descriptive nature of our data and the single time point assessed, this effect should not be interpreted as a direct mechanism underlying antidepressant action. Rather, it may reflect an early pharmacological modulation of neuronal responsiveness to stress.

In contrast to the IEGs, BDNF expression was not increased across all examined brain regions following swim stress. Stress-induced elevations were observed only in the striatum. In this region, only imipramine attenuated the stress-related increase in BDNF expression. Notably, the ketamine-treated group under no-stress conditions showed significantly higher BDNF levels compared with the other unstressed groups. The reason for this difference is unclear; however, it is reported here for transparency. Nevertheless, the high variability in striatal BDNF expression likely reflects the very low basal levels of BDNF mRNA in this region, as previously reported [33]. Moreover, these results are consistent with the temporal dynamics of BDNF, which typically peaks later after stimulation, and suggest that our 30 min post-swim stress window was optimal for IEG induction but may have been too early to capture robust BDNF regulation. Nevertheless, we included it in our analysis to assess whether early engagement of this plasticity-related pathway could already be detected following acute stress and pharmacological treatment. Moreover, assessing BDNF alongside IEGs allowed us to compare rapid transcriptional responses to neuronal activation with the early recruitment of a major plasticity-related factor.

An important aspect emerging from our data, even beyond the drug effects, is the distinct pattern of IEG induction following swim stress in vehicle-treated animals. This acute stressor triggered robust, yet gene-specific transcriptional responses across brain regions. In the hippocampus, swim stress induced a broad and consistent upregulation of *Npas4*, *Zif268*, and *c-Fos*, in line with the well-documented role of this region in stress response and plasticity [34]. In contrast, in the frontal cortex and striatum, the transcriptional profile was less pronounced. *c-Fos* exhibited consistent strong induction, whereas *Arc*, *Zif268* and *Npas4* displayed lower or no responses. These differences suggest that induced stress recruits partially distinct molecular programs depending on both gene identity and brain region. Indeed, although *Arc*, *Npas4*, *Zif268*, and *c-Fos* are all classified as IEGs due to their rapid and transient induction by neuronal activity, they are not interchangeable markers of activation but rather contribute to distinct aspects of synaptic function and circuit plasticity. Furthermore, whereas *Arc*, *c-Fos*, and *Zif268* largely converge as canonical markers of excitatory-driven synaptic plasticity, *Npas4* stands apart by orchestrating inhibitory circuit remodeling [8,35].

Several limitations of the present study should be acknowledged. First, we employed only male C57BL/6N mice, which limits the generalizability of our findings, as sex- and strain-dependent differences in behavioral and molecular responses to antidepressants could be present. Secondly, we focused on a single post-swim stress time point (30 min), which is optimal for IEG induction but may not adequately reflect the delayed transcriptional regulation of plasticity-related factors. Future studies incorporating multiple time points could provide a more comprehensive picture of the transcriptional cascades engaged by acute stress and antidepressant treatments. Finally, we limited our molecular analyses to a subset of brain regions. Although these areas are central to stress regulation and antidepressant action, additional brain structures such as the amygdala and habenula are also critically involved and could contribute to the differential mechanisms of ketamine and imipramine.

## 5. Conclusions

In conclusion, our study shows that both ketamine and imipramine increase latency to immobility during swim stress exposure, indicating a shared enhancement of the initial active coping response. However, only imipramine significantly reduces total immobility time, suggesting that the behavioral effects of the two treatments only partially overlap.

Imipramine exerts a broader suppression of cortical and striatal cFOS induction, besides inducing an additional sustained passive coping capacity. The hippocampus, despite being robustly activated by stress, appears less sensitive to acute pharmacological antidepressant modulation. The temporal dissociation between IEGs and BDNF further underscores the importance of capturing dynamic transcriptional processes at multiple time points. By integrating behavioral and molecular endpoints, our findings provide new insights into the shared and divergent mechanisms of classical and rapid-acting antidepressants, highlighting fronto-striatal activity-dependent plasticity as a key substrate of their acute effects. Eventually, our findings should be interpreted as describing how acute antidepressant administration modulates stress-evoked transcriptional responses and coping strategies rather than modeling antidepressant efficacy.

## Figures and Tables

**Table 1:** Mixed-model ANOVA (Type III tests) for the effects of Treatment, FST, Gene, and Region on Value. Mixed-effects model: Value ~ Treatment × FST × Gene × Region + (1|Subject).

Effect	df	F	*p*	Partial Eta-Sq
Treatment	2, 31.40	4.40	0.021	0.02
FST	1, 31.40	73.15	1.05 × 10^−9^	0.15
Gene	4, 403.58	85.39	1.70 × 10^−52^	0.45
Region	2, 412.45	64.95	3.00 × 10^−25^	0.24
Treatment:FST	2, 31.40	8.54	0.001	0.04
Treatment:Gene	8, 403.57	4.33	5.04 × 10^−5^	0.08
FST:Gene	4, 403.58	42.05	1.78 × 10^−29^	0.29
Treatment:Region	4, 412.20	3.31	0.011	0.03
FST:Region	2, 412.15	15.19	4.30 × 10^−7^	0.07
Gene:Region	8, 404.38	22.75	1.03 × 10^−28^	0.30
Treatment:FST:Gene	8, 403.57	5.05	5.46 × 10^−6^	0.09
Treatment:FST:Region	4, 412.40	10.67	3.09 × 10^−8^	0.09
Treatment:Gene:Region	16, 404.37	1.99	0.013	0.07
FST:Gene:Region	8, 404.38	4.66	1.83 × 10^−5^	0.08
Treatment:FST:Gene:Region	16, 404.37	4.51	2.74 × 10^−8^	0.15

## Data Availability

The original contributions presented in this study are included in the article/Supplementary Materials. For further inquiries, please contact the corresponding author.

## Supplementary Materials

The following supporting information can be downloaded at: https://www.mdpi.com/article/10.3390/biom16040484/s1, Figure S1: Experimental design; Table S1: Nucleotide sequences of primers and probes of TaqMan assays purchased from Eurofins Genomics (Vimodrone, Italy).

## Abbreviations

The following abbreviations are used in this manuscript:

MDD	Major Depressive Disorder
FST	Forced Swim Test
IEGs	Immediate Early Genes
BDNF	Brain-Derived Neurotrophic Factor
TrkB	Tropomyosin receptor kinase B
NMDA	N-methyl-D-aspartate
AMPA	α-amino-3-hydroxy-5-methyl-4-isoxazolepropionic acid
mTOR	Mammalian Target of Rapamycin
qRT-PCR	Quantitative Real-Time Polymerase Chain Reaction
SEM	Standard Error of the Mean
ANOVA	Analysis of Variance
