# Supplementary material for: Distinct Molecular Responses to Ketamine and Imipramine in Cortical and Striatal Regions Following Acute Swim Stress
Source: Biomolecules. 2026 Mar 24;16(4):484. doi: 10.3390/biom16040484 (PMC13114071; doi:10.3390/biom16040484)
Supplement: Supplementary file 1 [file biomolecules-16-00484-s001.zip › Supplementary Table S1.pdf]

Supplementary Table S1. Nucleotide sequences of primers and probes of TaqMan assays purchased from Eurofins Genomics (Vimodrone, Italy).

Gene	Forward Primer	Reverse Primer	Probe
Npas4	GTCCTAATCTACCTGGGCTTTG	TCTCCACTTTCAGCCAACAG	ATGGTATGGACTGCTACACCCCGA
Zif268	AGCGCCTTCAATCCTCAAG	TTTGGCTGGGATAACTCGTC	CAACCCTATGAGCACCTGACCACA
c-Fos	TCCTTACGGACTCCCCAC	CTCCGTTTCTCTTCCTCTTCAG	TGCTCTACTTTGCCCCTTCTGCC
Bdnf	AAGTCTGCATTACATTCCTCGA	GTTTTCTGAAAGAGGGACAGTTTAT	TGTGGTTTGTTGCCGTTGCCAAG
