# Supplementary figures and images for: Distinct Molecular Responses to Ketamine and Imipramine in Cortical and Striatal Regions Following Acute Swim Stress
Source: Biomolecules. 2026 Mar 24;16(4):484. doi: 10.3390/biom16040484 (PMC13114071; doi:10.3390/biom16040484)

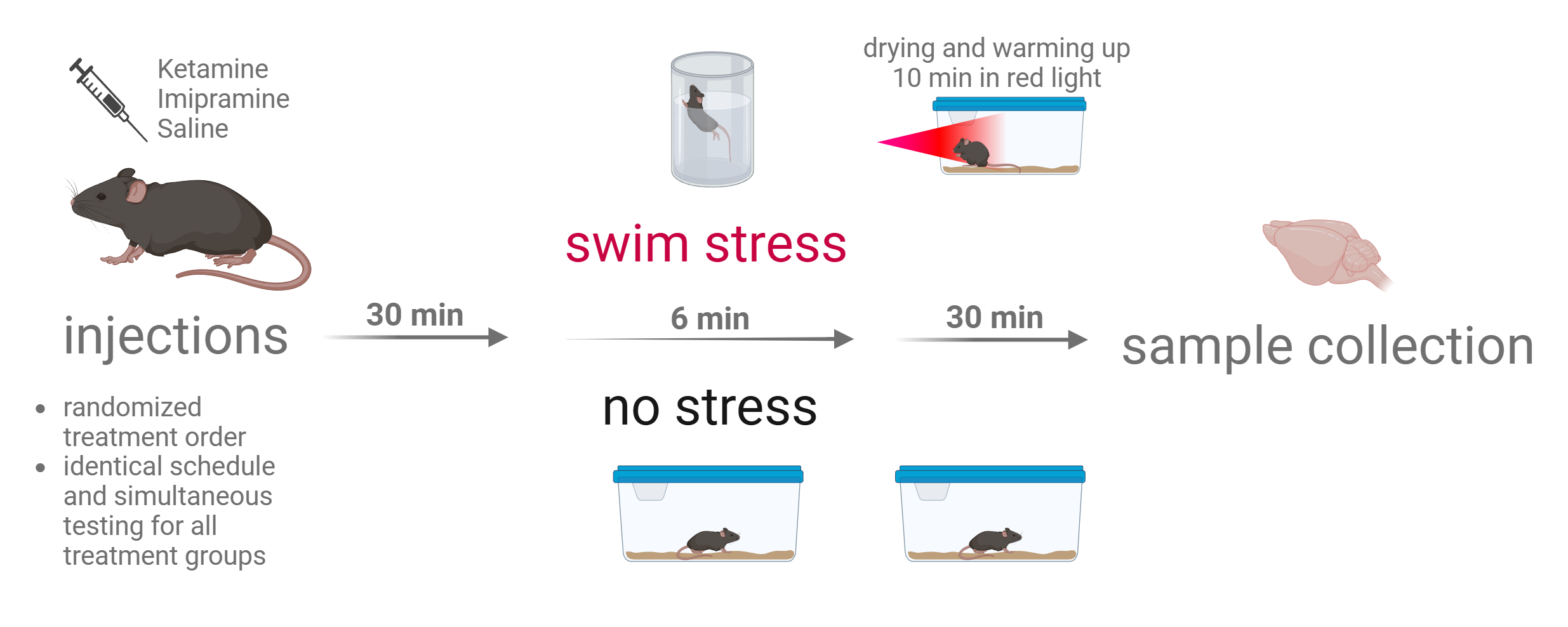

Supplement: Supplementary file 1 [file biomolecules-16-00484-s001.zip › Supplementary Figure S1.png]
